# Troubleshooting for difficult removal of a stent delivery system after endoscopic ultrasound-guided hepaticogastrostomy

**DOI:** 10.1055/a-2094-9374

**Published:** 2023-06-12

**Authors:** Takeshi Ogura, Taro Iwatsubo, Jun Sakamoto, Junichi Nakamura, Masahiro Yamamura, Hiroki Nishikawa

**Affiliations:** 1 Endoscopic Center, Osaka Medical and Pharmaceutical University Hospital, Osaka, Japan; 2 Second Department of Internal Medicine, Osaka Medical and Pharmaceutical University Hospital, Osaka, Japan

Endoscopic ultrasound-guided hepaticogastrostomy (EUS-HGS) is now widely performed for patients in whom endoscopic retrograde cholangiopancreatography is unsuccessful 1 2 3 4 . Various technical tips have been reported for preventing adverse events and improving the technical success rate before stent deployment. We herein describe a troubleshooting technique for difficult removal of the stent delivery system after EUS-HGS stent deployment.

A 77-year-old woman was admitted for treatment of obstructive jaundice due to unresectable hepatic hilar carcinoma. Multiple uncovered self-expandable metal stents (UCSEMSs) were inserted and systemic chemotherapy was attempted; however, recurrent biliary obstruction was observed after 6 months. Reintervention using UCSEMS was then performed for the left, anterior, and posterior bile ducts, but biliary obstruction recurred after 4 months. Further reintervention was successful for the right hepatic bile duct but failed for the left hepatic bile duct. Therefore, EUS-HGS was attempted.

The intrahepatic bile duct was punctured using a 19 G needle. After injection of contrast medium, a 0.025-inch guidewire was deployed. Tract dilation was performed using a drill dilator. An 8.5 Fr stent delivery system was inserted and the stent was successfully deployed (8 mm × 12 cm, Spring Stopper stent; Taewoong Medical, Seoul, South Korea) ( Fig. 1 a ). However, the stent delivery system could not be removed because of insufficient stent expansion ( Fig. 1 b ). Therefore, the stent delivery system was cut close to the patient’s mouth using pliers, and the echoendoscope was removed ( Fig. 2 ). A duodenoscope was inserted and a guidewire was inserted into the biliary tract through the EUS-HGS stent. After balloon dilation at the site of insufficient stent expansion ( Fig. 3 a ), the stent delivery system was removed successfully without any adverse events ( Fig. 3 b , Video 1 ).

**Fig. 1:**
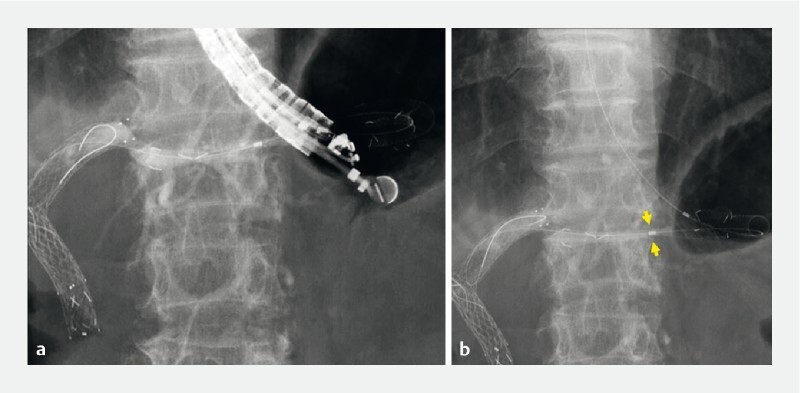
Endoscopic ultrasound-guided hepaticogastrostomy. **a** Stent deployment was successfully performed. **b** The stent delivery system could not be removed owing to insufficient stent expansion (arrows).

**Fig. 2:**
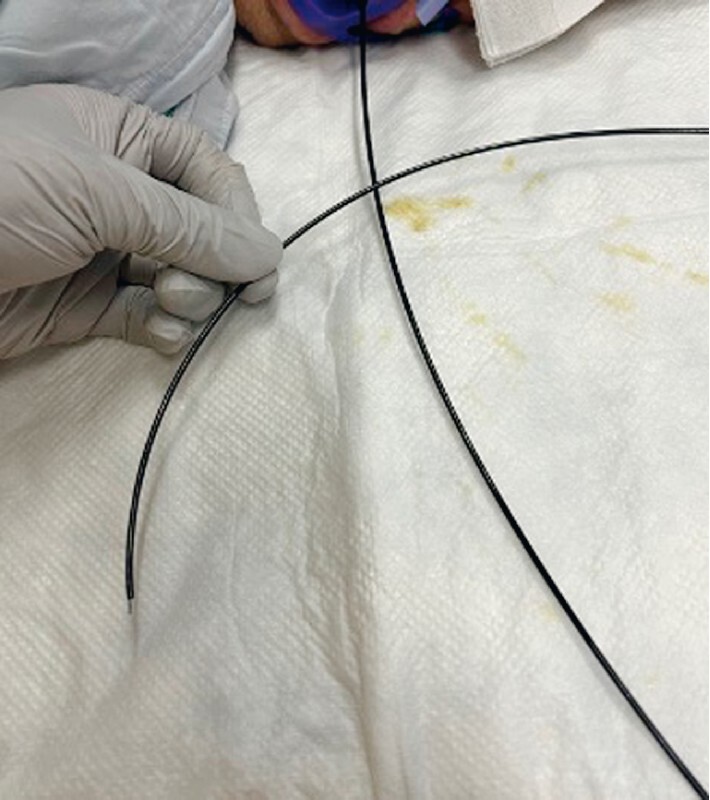
The stent delivery system was cut close to the patient’s mouth using pliers.

**Fig. 3:**
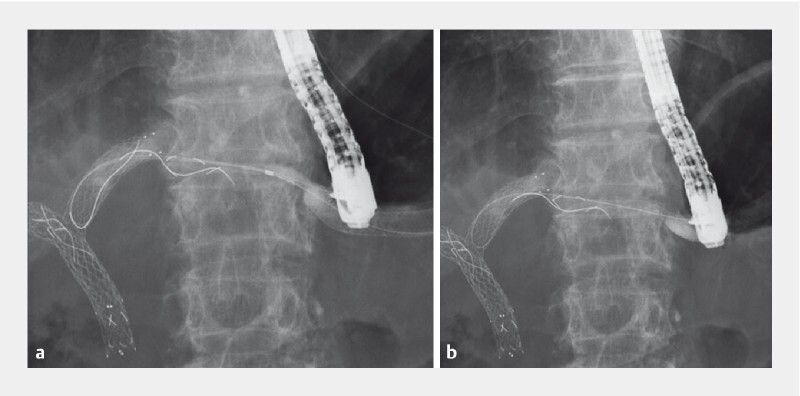
Removal of the delivery system. **a** After guidewire insertion, balloon dilation was performed at the site of insufficient stent expansion. **b** The stent delivery system was successfully removed.

**Video 1** Troubleshooting procedure in cases of difficult removal of a stent delivery system due to insufficient stent expansion.


In cases of difficult removal of a stent delivery system due to insufficient stent expansion, additional dilation at the site of insufficiency may enable removal of the system.

Endoscopy_UCTN_Code_CPL_1AL_2AD

## Notes

Endoscopy E-Videos

https://eref.thieme.de/e-videos
*E-Videos* is an open access online section of the journal *Endoscopy* , reporting on interesting cases and new techniques in gastroenterological endoscopy. All papers include a high-quality video and are published with a Creative Commons CC-BY license. Endoscopy E-Videos qualify for HINARI discounts and waivers and eligibility is automatically checked during the submission process. We grant 100% waivers to articles whose corresponding authors are based in Group A countries and 50% waivers to those who are based in Group B countries as classified by Research4Life (see: https://www.research4life.org/access/eligibility/ ). This section has its own submission website at https://mc.manuscriptcentral.com/e-videos
